# Salvage therapy containing sorafenib and donor lymphocyte infusion is associated with improved outcomes for FLT3 wild-type acute myeloid leukemia relapsing after allogeneic hematopoietic stem cell transplantation

**DOI:** 10.1186/s40364-025-00816-9

**Published:** 2025-08-20

**Authors:** Zhonghui Jiang, Ya Zhou, Yuxin Bai, Fang Dai, Menglin Fan, Tian Zhang, Danian Nie, Yunxin Zeng, Yirong Jiang, Ping Zhu, Zhiping Fan, Na Xu, Fen Huang, Ren Lin, Min Dai, Xiaojun Xu, Zhangkun Li, Hua Jin, Jing Sun, Qifa Liu, Li Xuan

**Affiliations:** 1 Department of Hematology, Nanfang Hospital, Southern Medical University, Guangzhou, 510515 China; 2 Clinical Medical Research Center of Hematology Diseases of Guangdong Province, Guangzhou, China; 3 Department of Hematology, Sun Yat-sen Memorial Hospital, Sun Yat-sen University, Guangzhou, China; 4 Department of Hematology, Seventh Affiliated Hospital of Sun Yat-Sen University, Shenzhen, China; 5 Department of Hematology, Dongguan People’s Hospital, Southern Medical University, Dongguan, China; 6 Department of Hematology, First People’s Hospital of Chenzhou, Chenzhou, China

**Keywords:** Sorafenib, Relapse after allogeneic hematopoietic stem cell transplantation, Salvage therapy, FLT3 wide-type

## Abstract

**Background:**

There is no established standard treatment for acute myeloid leukemia (AML) patients with FLT3 wild-type relapsing after allogeneic hematopoietic stem cell transplantation (allo-HSCT). Multi-kinase inhibitor sorafenib has been widely explored in the treatment of AML patients with FLT3 internal tandem duplication (FLT3-ITD) mutations. Some studies have revealed that the addition of sorafenib to standard chemotherapy could improve outcomes in newly diagnosed AML regardless of FLT3 status. However, the application of sorafenib in FLT3 wild-type AML patients experiencing relapse after allo-HSCT remains minimally investigated.

**Methods:**

We retrospectively compared the effects of conventional treatment combined with or without sorafenib on the outcomes of these patients. The study mainly focused on the treatment response of salvage therapy and survival.

**Results:**

Sixty-two AML patients with FLT3 wild-type who relapsed after allo-HSCT were enrolled in this study, including 38 with sorafenib and 24 without sorafenib. Fifty patients received 68 doses of donor lymphocyte infusion (DLI). The rate of composite complete remission was 65.8% in the sorafenib group, compared with 29.2% in the non-sorafenib group (*P* = 0.005). With a median follow-up of 13.2 months (IQR, 3.2–43.5) after relapse, the 2-year overall survival (OS) was 47.4% (95%CI, 33.9%-66.2%) and 16.7% (6.8%-40.8%) in the sorafenib and non-sorafenib groups (*P* = 0.006). The 2-year event-free survival (EFS) was 44.7% (31.4%-63.7%) and 16.7% (6.8%-40.8%) in the two groups (*P* = 0.012). Multivariable analysis revealed that salvage therapy including sorafenib was the protective factor for longer OS and EFS (HR = 0.395, 95% CI: 0.209–0.746, *P* = 0.004; HR = 0.406, 95% CI: 0.218–0.754, *P* = 0.004). The incidences of acute graft-versus-host disease (GVHD) and chronic GVHD were similar between the two groups (*P* = 0.806, *P* = 0.908).

**Conclusion:**

Our results suggest that salvage therapy including sorafenib and DLI is associated with improved outcomes for AML patients with FLT3 wild-type relapsing after allo-HSCT.

## Background

Allogeneic hematopoietic stem cell transplantation (allo-HSCT) is an effective therapy for acute myeloid leukemia (AML). However, post-transplant relapse occurs in a considerable proportion of patients. Patients who experience leukemia relapse post-transplantation have a poor prognosis, with the 2-year overall survival (OS) of only 20% [1–3]. To date, there is no established standard treatment for post-transplant relapse. Conventional therapeutic options include chemotherapy, donor lymphocyte infusion (DLI), and second allo-HSCT. Despite such treatments, however, the efficacy is limited [1–3].

Currently, the multi-kinase inhibitor sorafenib has been widely explored in the treatment of AML [4–14]. Sorafenib maintenance post-transplantation has been recommended as first-line treatment in AML patients with FLT3 internal tandem duplication (FLT3-ITD) mutations by multiple guidelines [15, 16]. Several studies have revealed that the addition of sorafenib to standard chemotherapy could result in improved survival in newly-diagnosed AML, irrespective of the FLT3-mutation status [4, 11, 12]. However, there are few studies on the use of sorafenib in AML patients with FLT3 wild-type who relapse after allo-HSCT. Our previous study has demonstrated that sorafenib combined with chemotherapy and DLI is also effective for patients with FLT3 wild-type AML relapsing after allo-HSCT [10]. Apart from targeting multiple kinases, growing evidence has shown that the antitumor effect of sorafenib is related to immunomodulatory activity [17, 18]. Considering the enhancement effect of sorafenib on graft-versus-leukemia (GVL), we conducted a retrospective exploratory study to compare conventional therapeutic strategies combined with or without sorafenib for AML patients with FLT3 wild-type who relapsed after allo-HSCT.

## Methods

### Patients and data collection

This retrospective study examined all consecutive AML patients with FLT3 wild-type who experienced hematologic and/or extramedullary relapse after allo-HSCT at 5 hospitals (Nanfang Hospital, Sun Yat-sen Memorial Hospital, Seventh Affiliated Hospital of Sun Yat-sen University, Dongguan People’s Hospital and First People’s Hospital of Chenzhou) from November 2017 to December 2022. In our five centers, molecular biology including FLT3 mutation status was evaluated at the initial diagnosis. Most patients did molecular genetics analysis including FLT3 mutation status at relapse both pre-transplant and post-transplant. Patients were eligible for this study if they were FLT3 wild-type at the initial diagnosis and at relapse after allo-HSCT. Patients were excluded from the study if they gave up further treatment after relapse post-transplant.

Data were obtained from patients’ medical records. Variables collected for all patients included demographic information, pre-transplant-related parameters, transplant-related parameters, post-transplant-related parameters, relapse-related parameters post-transplant, treatment-related parameters, and survival. The trial was first approved by Medical Ethics Committee of Nanfang Hospital of Southern Medical University. Subsequently, the remaining four centers conducted ethical filing. This trial was reviewed and approved by the ethics committee review board at each participating center and was conducted in accordance with the principles of the Declaration of Helsinki. Written informed consent was obtained from all the patients.

### Measurable residual disease (MRD) detection

Generally, bone marrow (BM) was evaluated at 1, 2, 3, 4, 6, 8, 10, and 12 months within 1-year post-transplantation, then at 3-month intervals during second year, 4-month intervals within third year, and half-year intervals from fourth to fifth year. MRD was evaluated by eight-color multi-parameter flow cytometry (MFC) with a threshold of 0.01%. MRD was also monitored by quantitative polymerase chain reaction (PCR) for leukemia-related specific genes including RUNX1-RUNX1T1and CBFβ/MYH11, with a threshold of 0.001%. Patients were defined as MRD positivity if they had two consecutive positive results using MFC or PCR or were both positive in a single sample. Preemptive therapy was conducted in patients with MRD positive post-transplant.

### Evaluation points and definitions

This study mainly focused on the treatment response of salvage therapy and survival. Leukemia relapse was defined as either reappearance of leukemic blasts in the peripheral blood or at least 5% blasts in the BM aspirate or biopsy specimen not attributable to any other cause, or reappearance or new appearance of extramedullary leukemia. Response criteria were defined according to the European Leukemia Net (ELN) 2022 guidelines [15]. Complete remission (CR) was defined as BM blasts less than 5%, absence of circulating blasts and absence of extramedullary disease, and an absolute neutrophil count of 1.0 × 10⁹ cells/L or higher and a platelet count of 100 × 10⁹/L or higher. Complete remission with incomplete hematological recovery (CRi) met all criteria for CR except that for residual neutropenia (absolute neutrophil count < 1.0 × 10⁹ cells/L), thrombocytopenia (platelets < 100 × 10⁹ cells/L) or both. Composite complete remission (CRc) comprised CR and CRi. Non-remission (NR) was defined as a failure to obtain CRc.

OS was defined as the time from the initiation of salvage therapy until death or last follow-up. Event-free survival (EFS) was defined as the time from salvage therapy until documented failure to achieve CRc, relapse after CRc, or death from any cause, whichever occurred first. Acute graft-versus-host disease (aGVHD) was graded according to the 1994 Consensus Conference on Acute GVHD Grading [19], and chronic GVHD (cGVHD) was graded according to the National Institutes of Health criteria [20]. The timing of GVHD before relapse was defined as the interval from HSCT to the onset of GVHD. The timing of GVHD after relapse was defined as the interval from the start of salvage therapy to the onset of GVHD.

### Statistical analysis

The study data were analyzed on December 31, 2024. The descriptive analysis of patient characteristics included median and interquartile range (IQR) for continuous variables, and absolute and relative frequencies for categorical variables. The χ² test was used to assess categorical variables and the Mann-Whitney U test was used for continuous variables. The CRc rate and two-sided 95% confidence intervals (CIs) were calculated with the Wilson method. OS and EFS were analyzed by the Kaplan-Meier method and compared using the log-rank test. The corresponding hazard ratio (HR) and 95% CI were estimated using the Cox proportional hazards model. Cumulative incidence of GVHD was calculated by accounting for competing risk. Death due to other causes was considered a competing event for GVHD. The comparison of the cumulative incidence in the presence of a competing risk was done using the Fine and Gray model [21]. The Cox proportional hazards model was used for the analysis of risk factors for time-to-event variables. The test indicated that the proportional hazards assumptions held. Variables included in the univariable analysis were gender, age, disease status at transplant, donor type, ELN classification, relapse type post-transplant, relapse time post-transplant, salvage therapy including sorafenib, salvage therapy including DLI, and acute and chronic GVHD after salvage therapy. Only variables with a p value less than 0.10 were included in the multivariable analysis. All statistical tests were two-tailed with a significance level of 0.05. SPSS version 25.0 and R version 3.3.0 were used for data analysis.

## Results

### Patient characteristics

Except for the 14 patients who abandoned further treatment after relapse, 62 AML patients with FLT3 wild-type who relapsed after allo-HSCT were enrolled in this study. The median age at the time of transplant was 35.5 years old (IQR, 29 to 48.5), and there were 42 male and 20 female. With respect to ELN 2022 risk, 4 (6.5%), 14 (22.6%), and 44 (71.0%) patients were favorable-, intermediate-, and adverse-risk, respectively. Forty patients were in first complete remission, 5 in ≥ second complete remission, and 17 in NR at the time of transplant. Thirty-two patients received HLA-matched sibling donor and 30 received HLA-haploidentical related donor transplant. The 62 patients all achieved CRc and complete chimerism by day + 30 post-transplant.

With a median time of 192.5 days (IQR, 94.0-397.5) after allo-HSCT, 47 patients experienced hematologic relapse, 7 extramedullary relapse, and 8 hematologic accompanied by extramedullary relapse. Based on sorafenib inclusion in salvage therapy, patients were divided into two groups: sorafenib (*n* = 38) and non-sorafenib (*n* = 24). The baseline factors of patients’ characteristics were well balanced between the two groups (Table 1). None of the patients received any FLT3 inhibitors other than sorafenib for salvage treatment. There was also no history of sorafenib use prior to relapse after transplantation.

**Table 1:** Patients’ clinical and transplant characteristics

	Entire sample (*n* = 62)	Sorafenib Group (*n* = 38)	Non-sorafenib Group (*n* = 24)	*P*
Median age at transplant, yr (IQR)	35.5(29-48.5)	33(28.6–47.8)	37(32.3–51)	0.424
Gender				0.331
Male	42(67.7%)	24(63.2%)	18(75.0%)	
Female	20(32.3%)	14(36.8%)	6(25.0%)	
ELN classification^#^, No (%)				0.811
Favorable	4(6.5%)	2(5.3%)	2(8.3%)	
Intermediate	14(22.6%)	8(21.1%)	6(25.0%)	
Adverse	44 (71.0%)	28(73.7%)	16(66.7%)	
Disease status at transplant				0.692
CR1	40(64.5%)	26(68.4%)	14(58.3%)	
≥CR2	5(8.1%)	3(7.9%)	2(8.3%)	
NR	17(27.4%)	9(23.7%)	8(33.4%)	
Donor type				0.213
HLA-matched sibling	32(51.6%)	22(57.9%)	10(41.7%)	
HLA-haploidentical related	30(48.4%)	16(42.1%)	14(58.3%)	
Median relapse time post-transplant, days (IQR)	192.5 (94-397.5)	192.5 (92.3–476.0)	194.5 (104.3–314.0)	0.426
aGVHD before relapse				0.715
No aGVHD	43(69.4%)	27(71.1%)	16(66.7%)	
aGVHD	19(30.6%)	11(28.9%)	8(33.3%)	
cGVHD before relapse				0.984
No cGVHD	49(79.0%)	30(78.9%)	19(79.2%)	
cGVHD	13(21.0%)	8(21.1%)	5(20.8%)	
Relapse type post-transplant				0.971
Hematologic	47(75.8%)	29(76.3%)	18(75.0%)	
Extramedullary	7(11.3%)	4(10.5%)	3(12.5%)	
Hematologic + extramedullary	8(12.9%)	5(13.2%)	3(12.5%)	

Data are n (%) or median (IQR). Percentages might not total 100 because of rounding. ELN, European Leukemia Net; CR1, first complete remission; CR2, second complete remission; NR, no response; aGVHD, acute graft-versus-host disease; cGVHD, chronic graft-versus-host disease. ^#^referred to 2022 European Leukemia Net risk stratification

### Salvage therapies and response

Once patients relapsed after allo-HSCT, immunosuppressants were withdrawn/ stopped immediately, and salvage therapy was taken. Four salvage regimens were administered after relapse post-transplant, including sorafenib combined with chemotherapy followed by DLI (*n* = 33), sorafenib combined with chemotherapy (*n* = 5), chemotherapy followed by DLI (*n* = 17) and monochemotherapy (*n* = 7). Besides, 5 patients with extramedullary relapse also received radiotherapy.

Thirty-eight patients were treated with sorafenib for a median duration of 360 days (IQR, 121 to 522) after relapse post-transplant. Sorafenib was usually used at the beginning of induction chemotherapy. The initial dose of sorafenib was 400 mg twice daily and adjusted based on suspected toxicity (dose range, 200 to 800 mg daily). The chemotherapy regimens included venetoclax, homoharringtonine and azacytidine (VAH) regimen (*n* = 30), venetoclax and azacytidine (VA) regimen (*n* = 6), decitabine, aclacinomycin, cytarabine and granulocyte colony-stimulating factor (DAC-CAG) regimen (*n* = 15) and other regimens (*n* = 11). For patients without grades II to > II aGVHD or extensive cGVHD at the time of relapse, granulocyte colony-stimulating factor (G-CSF)-mobilized DLI was administered at the following day of chemotherapy end if donor lymphocytes were available. Eighty-eight DLI doses were administered to 50 patients, with a median of once per patient (IQR, 1 to 2) and a median dosage of 3.0 × 10^7^ CD3^+^ T cells/kg (IQR, 2.5 to 4.3).

Thirty-eight patients in the sorafenib group received chemotherapy regimens including VAH regimen (*n* = 22), VA regimen (*n* = 4), DAC-CAG regimen (*n* = 7) and other regimens (*n* = 5). Twenty-four patients in the non-sorafenib group received chemotherapy regimens including VAH regimen (*n* = 8), VA regimen (*n* = 2), DAC-CAG regimen (*n* = 8) and other regimens (*n* = 6). In the sorafenib group, 54 doses of DLI were administered in 33 of 38 patients, with a median of once per patient (IQR, 1 to 2). In the non-sorafenib group, thirty-four doses of DLI were administered to 17 of 24 patients, with a median of once per patient (IQR, 1 to 3).

**Fig. 1:**
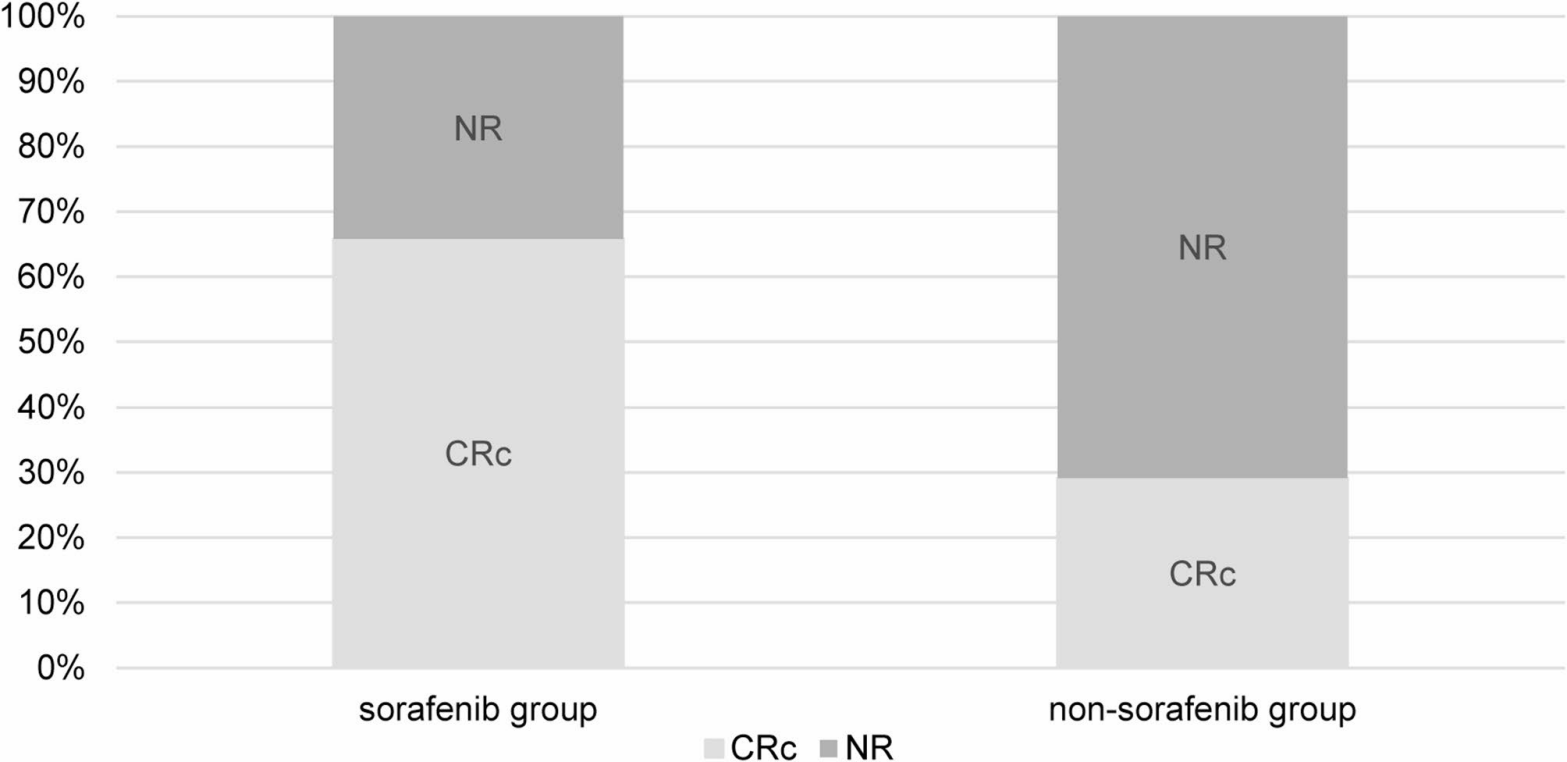
The response of patients who relapsed post-transplant receiving salvage therapy with or without sorafenib. CRc, composite complete remission; NR, no response

Thirty-two patients achieved CRc after salvage therapy, with the CRc rate of 51.6% (32 of 62 patients; 95% CI, 39.5%-63.6%). The CRc rate was 65.8% (25 of 38 patients; 95% CI, 49.9%-78.8%) in the sorafenib group, compared with 29.2% (7 of 24 patients; 95% CI, 14.9%-49.2%) in the non-sorafenib group (*P* = 0.005, Fig. 1, Table 2).

**Table 2:** Outcomes after salvage therapy

	Entire sample (*n* = 62)	Sorafenib Group (*n* = 38)	Non-sorafenib Group (*n* = 24)	*P*
CRc rate, No. (% [95% CI])	32 (51.6 [39.5–63.6])	25(65.8 [49.9–78.8])	7(29.2 [14.9–49.2])	*0.005
OS				
Median, months (95% CI)	12.8(3.2–22.5)	18.4(0-50.8)	4.8(2.0-7.6)	
1-year OS, % (95% CI)	51.6(40.6–65.7)	63.2(49.5–80.5)	33.3(18.9–58.7)	*0.016
Estimated 2-year OS, % (95% CI)	35.5(25.4–49.6)	47.4(33.9–66.2)	16.7(6.8–40.8)	*0.006
EFS				
Median, months (95% CI)	4.3(0-11.4)	11.4(0-32.2)	2.3(0.2–4.3)	
1-year EFS, % (95% CI)	40.3(29.8–54.6)	50.0(36.4–68.7)	25.0(12.5–50.0)	*0.037
Estimated 2-year EFS, % (95% CI)	33.9(23.9–48.0)	44.7(31.4–63.7)	16.7(6.8–40.8)	*0.012

Abbreviations: CRc, composite complete remission; OS, overall survival; EFS, event-free survival; * *P*<0.05

Thirty-two patients received a median of 2 (IQR, 1-2.5) cycles of consolidation chemotherapy after CRc. In the sorafenib group, 25 patients with CRc received sorafenib maintenance for a median duration of 421 days (IQR, 343 to 617) after CRc. The median relative dose intensity of sorafenib maintenance was 400 (IQR, 400–800) mg/day. Eight of the 25 patients with CRc received sorafenib combined with interferon-ɑ (IFN-ɑ) as maintenance therapy. IFN-ɑ was administered subcutaneously at a dosage of 3 million units 2–3 times per week. The median duration of IFN-ɑ maintenance was 165 (IQR, 97.5 to 225) days. One patient in the sorafenib group received a second allo-HSCT after achieving CR. One patient in the non-sorafenib group proceeded to a second allo-HSCT after achieving PR.

Of the 32 patients who achieved CRc after salvage therapy, 9 experienced re-relapse, including 6 in the sorafenib group and 3 in the non-sorafenib group. In the sorafenib group, 19 patients remained in CRc, 3 patients re-relapsed during sorafenib maintenance after CRc, and 3 patients re-relapsed at 3, 5, and 12 months after sorafenib was discontinued. The leukemia re-relapse rate did not differ significantly between sorafenib and non-sorafenib groups (15.8% vs. 12.5%, *P* = 0.898).

### Survival

**Fig. 2:**
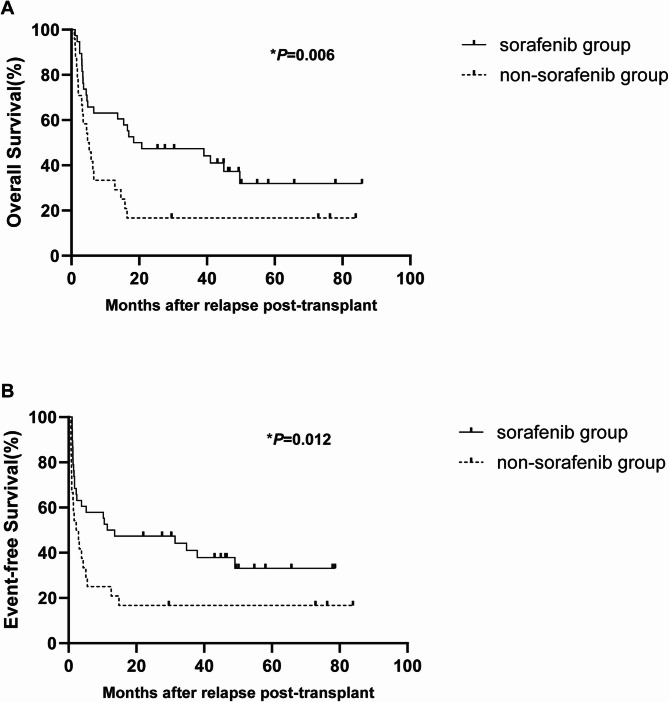
The outcomes of patients who relapsed post-transplant receiving salvage therapy with or without sorafenib. OS (**A**) and EFS (**B**) between the sorafenib and non-sorafenib groups

**Fig. 3:**
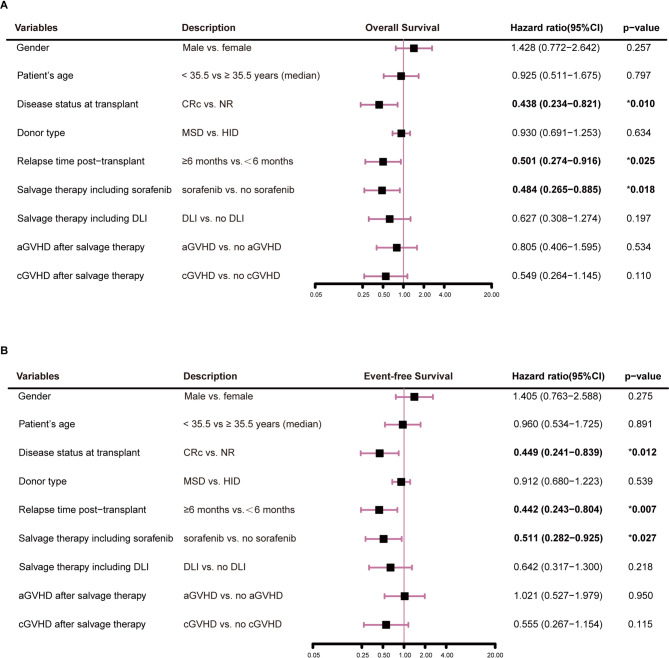
Forest plot for univariable analysis of overall survival (**A**) and event-free survival (**B**). CRc, composite complete remission; NR, no response; MSD, HLA-matched sibling donor; HID, HLA-haploidentical related donor; DLI, donor lymphocyte infusion; aGVHD, acute graft-versus-host disease; cGVHD, chronic graft-versus-host disease

With a median follow-up of 13.2 months (IQR, 3.2–43.5) after relapse, 18 patients were living and 44 deceased. Causes of death included leukemia progression (*n* = 34), infections (*n* = 9) and GVHD (*n* = 1). The 2-year OS and EFS were 35.5% (95% CI, 25.4%-49.6%) and 33.9% (23.9%-48.0%), respectively. The 2-year OS was 47.4% (95%CI, 33.9%-66.2%) for patients in the sorafenib group and 16.7% (6.8%-40.8%) in the non-sorafenib group (HR = 0.484, 95% CI: 0.265–0.885; *P* = 0.006; Fig. 2A); median OS was 18.4 months (95% CI, 0-50.8) and 4.8 months (2.0-7.6) in the sorafenib and non-sorafenib groups (Table 2). The 2-year EFS was 44.7% (31.4%-63.7%) for patients in the sorafenib group and 16.7% (6.8%-40.8%) in the non-sorafenib group, respectively (HR = 0.511, 95% CI: 0.282–0.925; *P* = 0.012; Fig. 2B); median EFS was 11.4 months (95% CI, 0-29.2) and 2.3 months (0.2–4.3) in the sorafenib and non-sorafenib groups (Table 2). Forest plot for univariable analysis of OS and EFS is shown in Fig. 3. The 2-year OS and EFS were 51.5% (95%CI, 37.0%-71.7%) and 48.5% (34.1%-68.9%) for patients in the group of sorafenib combined with chemotherapy and DLI, and 17.2% (7.8%-38.3%) and 17.2% (7.8%-38.3%) for patients in the other three groups, respectively (*P* = 0.003, *P* = 0.006). The 2-year OS was 51.5% (95%CI, 37.0%-71.7%) in the group of sorafenib combined with chemotherapy and DLI, and 20.0% (3.5%-100.0%) in the group of sorafenib combined with chemotherapy (*P* = 0.046). The 2-year EFS was 48.5% (34.1%-68.9%) and 20.0% (3.5%-100.0%) in the two groups (*P* = 0.035).

Multivariable analysis revealed that salvage therapy including sorafenib and CRc at transplant were protective factors for longer OS (HR = 0.395, 95% CI: 0.209–0.746, *P* = 0.004; HR = 0.493, 95% CI: 0.244–0.994, *P* = 0.048). Salvage therapy including sorafenib was a protective factor for longer EFS (HR = 0.406, 95% CI: 0.218–0.754, *P* = 0.004) (Table 3).

**Table 3:** Univariable and multivariable analyses for the risk factors of survival

Parameters	OS			EFS			
	Univariable HR (95%CI); *P*	Multivariable HR (95%CI); *P*		Univariable HR (95%CI); *P*	Multivariable HR (95%CI); *P*		
Gender male vs. female	1.428 (0.772–2.642); 0.257	-		1.405 (0.763–2.588); 0.275	**-**		
Patient’s age < 35.5 vs. ≥ 35.5 years (median)	0.925 (0.511–1.675); 0.797	-		0.960 (0.534–1.725); 0.891	**-**		
Disease status at transplant CRc vs. NR	0.438 (0.234–0.821); *0.010	0.493 (0.244–0.994) *0.048		0.449 (0.241–0.839); *0.012	0.566 (0.283–1.131); 0.089		
Donor type							
HLA-matched sibling vs. HLA-haploidentical related	0.930 (0.691–1.253); 0.634	-		0.912 (0.680–1.223); 0.539	**-**		
ELN classification^#^							
Adverse	Reference	-		Reference	-		
Intermediate	0.600 (0.278–1.297); 0.194	-		0.557 (0.257–1.207); 0.138	-		
Favorable	0.427 (0.102–1.780); 0.243	-		0.405 (0.097–1.691); 0.215	-		
Relapse type post-transplant							
Hematologic	Reference	-		Reference	-		
Extramedullary	0.257 (0.061–1.072); 0.062	0.291 (0.065-1.300); 0.106		0.229 (0.055–0.952); *0.043	0.287 (0.065–1.269); 0.100		
Hematologic + extramedullary	0.699 (0.292–1.670); 0.420	-		0.716 (0.301–1.705); 0.451	-		
Relapse time post-transplant ≥6 months vs.<6 months	0.501 (0.274–0.916); *0.025	0.778 (0.396–1.532); 0.468		0.442 (0.243–0.804); *0.007	0.611 (0.313–1.195); 0.150		
Salvage therapy including sorafenib Sorafenib vs. no sorafenib	0.484 (0.265–0.885); *0.018	0.395 (0.209–0.746); *0.004		0.511 (0.282–0.925); *0.027	0.406 (0.218–0.754); *0.004		
Salvage therapy including DLI DLI vs. no DLI	0.627 (0.308–1.274); 0.197	-		0.642 (0.317-1.300); 0.218	**-**		
aGVHD after salvage therapy aGVHD vs. no aGVHD	0.805 (0.406–1.595); 0.534	-		1.021 (0.527–1.979); 0.950	-		
cGVHD after salvage therapy cGVHD vs. no cGVHD	0.549 (0.264–1.145); 0.110	-		0.555 (0.267–1.154); 0.115	**-**		

Abbreviations: OS, overall survival; EFS, event-free survival; HR, hazard ratios; CI, confidence interval; CRc, composite complete remission; NR, no response; DLI, donor lymphocyte infusion; aGVHD, acute graft-versus-host disease; cGVHD, chronic graft-versus-host disease; * *P*<0.05. ^#^referred to 2022 European Leukemia Net risk stratification

**Fig. 4:**
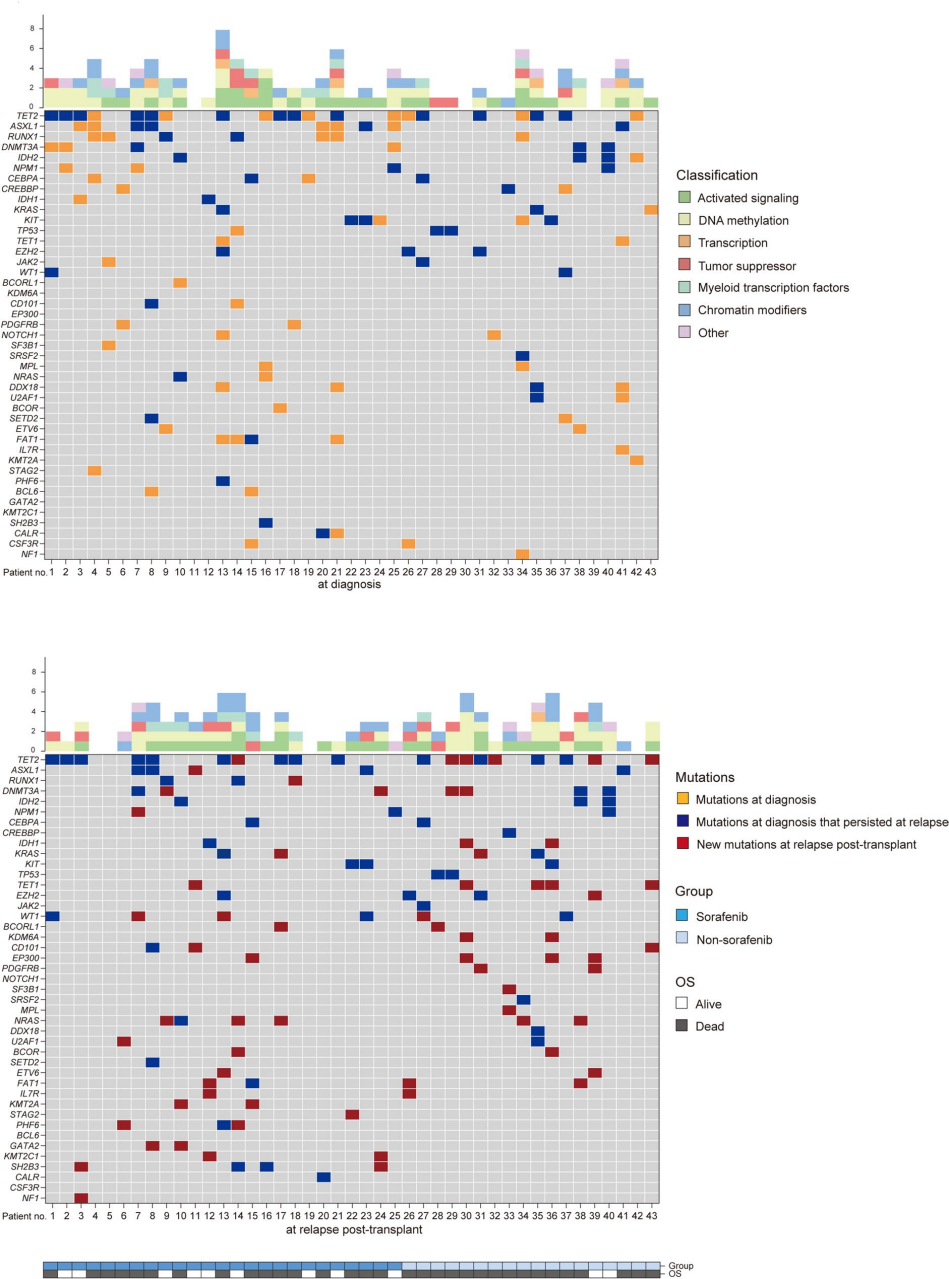
Mutations detected at diagnosis and at relapse in patients who relapsed post-transplant. OS, overall survival

Of the 62 AML patients, 43 patients were assessed for mutations at relapse post-transplant. The genetic landscape of the 43 patients at diagnosis and at relapse is shown in Fig. 4. Overall, 30 (69.8%) of the 43 evaluable relapsed patients had new gene mutations at relapse. The top three types of new mutations detected were activated signaling (13 patients, 30.2%), chromatin modifiers (13 patients, 30.2%) and DNA methylation (11 patients, 25.6%), respectively. The most frequently genes in new mutations were TET2 (6 patients, 14.0%), NRAS (5 patients, 11.6%) and TET1 (5 patients, 11.6%).

### Adverse events (AEs)

Because systemic chemotherapy might cause cytopenia, hematological AEs were not analyzed in this study. With a median of 14 days (range, 5–63 days) after sorafenib initiation, 15 patients (39.5%) experienced treatment-related AEs, including rash (*n* = 6), hand-foot-skin reaction (*n* = 4), diarrhea (*n* = 3), hypertension (*n* = 1), and abnormal liver enzymes (*n* = 1). Seven patients required sorafenib dose modifications because of AEs, including 5 dose reductions and 2 dose interruptions. With sorafenib dose modifications and symptomatic treatment, AEs were controlled and patients were re-administered sorafenib.

Nineteen patients developed aGVHD (grade I, *n* = 6; grade II, *n* = 10; grade III, *n* = 2; grade IV, *n* = 1), and 13 had cGVHD (local, *n* = 9; extensive, *n* = 4) before relapse. The 100-day cumulative incidence of aGVHD before relapse was 30.6% (95% CI, 19.6% -42.4%), and the 2-year cumulative incidence of cGVHD before relapse was 21.0% (11.8%-32.0%). Seventeen patients developed aGVHD (grade I, *n* = 7; grade II, *n* = 7; grade III, *n* = 2; grade IV, *n* = 1), and 16 had cGVHD (local, *n* = 11; extensive, *n* = 5) after salvage therapy. The 100-day cumulative incidence of aGVHD after salvage therapy was 27.4% (95% CI, 16.9%-39.0%), and the 2-year cumulative incidence of cGVHD was 21.4% (12.0%-32.7%), respectively. The incidences of aGVHD and cGVHD were similar between the patients in the sorafenib and non-sorafenib groups (*P* = 0.806, *P* = 0.908). The incidences of aGVHD and cGVHD did not differ significantly between the patients undergoing and those not undergoing DLI (*P* = 0.408, *P* = 0.359). The mortality of GVHD after salvage therapy was 1.6% (95% CI, 0.3%-8.6%).

## Discussion

The prognosis of AML patients with FLT3 wild-type relapsing after allo-HSCT is dismal [1–3]. Until now, there has been no standard approach to solve this difficult clinical problem. Multi-kinase inhibitor sorafenib has been used in a variety of settings for FLT3-ITD AML, including induction, post-remission maintenance, and salvage therapy [5–10, 13, 14]. Recent studies including our previous phase 2 trial have demonstrated that use of sorafenib might benefit AML patients with FLT3 wild-type [4, 11, 12, 22]. Currently, there is a lack of studies on the use of sorafenib in AML patients with FLT3 wild-type who relapse after allo-HSCT. In this exploratory study, we compared the efficacy of salvage therapy with and without sorafenib in AML patients with FLT3 wild-type relapsing after allo-HSCT, and found that salvage therapy containing sorafenib was superior to that without sorafenib with respect to response rates and survival. Twenty-five (65.8%) of the 38 patients in the sorafenib group achieved CRc, and the 2-year OS was 47.4%. For patients who relapsed after allo-HSCT, the CRc rate was reported ranging from 15 to 47% after conventional therapies, with the 2-year OS of only 26% [2, 3, 23]. Our results of CRc and OS were superior to those reported in the literature [2, 3, 23], although comparisons between different trials required to be made with caution. The superior efficacy might be attributed to the synergistic effect of sorafenib, chemotherapy and DLI to achieve a higher CRc rate and deep remission, as well as the superior OS obtained from post-remission maintenance therapy with sorafenib, consolidation chemotherapy and IFN-ɑ.

Except for blocking multiple pathways involved in the development of AML, sorafenib might also exert anti-leukemic effects through immune pathways [10, 17, 18]. Mathew et al. reported that sorafenib promoted GVL activity in mice and humans through interleukin-15 (IL-15) production in FLT3-ITD-mutant leukemia cells, resulting in activation of the IRF7-IL-15 axis and metabolic reprogramming of leukemia-reactive T cells [17]. In this study, sorafenib therapy was associated with improved outcomes for AML patients with FLT3 wild-type relapsing after allo-HSCT and the incidences of acute and chronic GVHD did not differ between patients with and without sorafenib. These results suggested that the effects of sorafenib were at least in part related to off-target immunomodulatory activity, and sorafenib inducing GVL effects might be independent of GVHD. The underlying mechanism requires further investigation. Besides, to evaluate the efficacy of sorafenib on FLT3-negative acute leukemia, we are doing a multi-center, randomized, phase 3 trial of sorafenib maintenance after allo-HSCT in patients with FLT3-negative acute leukemia (NCT04674345).

DLI is often associated with a high incidence and mortality of GVHD [24–26]. The incidences of aGVHD and cGVHD after DLI are reported to be 18.2%-56.9% and 16.6%-48.3%, respectively [24–26]. Whether the use of sorafenib post-transplantation increases GVHD is inconclusive [8, 10, 27]. In the current study, salvage therapy mainly included sorafenib combined with chemotherapy and DLI, and the incidences of aGVHD and cGVHD after salvage therapy were 27.4% and 21.4%. Our results were consistent with most literature reports about DLI [24–26], suggesting that sorafenib combined with DLI did not increase the risk of GVHD compared with DLI. These results suggested that our salvage therapy strategy was safe and welltolerated for AML patients with FLT3 wild-type who relapsed after allo-HSCT.

Our study had a few limitations. First, due to the retrospective study, the heterogeneity of the population including several centers and inherent selection bias was inevitable. Well-designed prospective clinical trials are needed to establish the optimal therapy for these relapsed patients. Second, the number of patients was relatively small, and some patients were followed for a short time, which might affect the accuracy of our results.

In conclusion, salvage therapy including sorafenib and DLI is associated with improved outcomes for AML patients with FLT3 wild-type who relapse after allo-HSCT. Sorafenib combined with chemotherapy and DLI could be a therapeutic option for FLT3 wild-type AML relapsing after allo-HSCT.

## Data Availability

The data that support the findings of this study are available from the corresponding author at 356135708@qq.com upon reasonable request.

## Abbreviations

AE: Adverse event
aGVHD: Acute graft-versus-host disease
allo-HSCT: Allogeneic hematopoietic stem cell transplantation
AML: Acute myeloid leukemia
BM: Bone marrow
cGVHD: Chronic graft-versus-host disease
CI: Confidence interval
CR: Complete remission
CRc: Composite complete remission
CRi: Complete remission with incomplete hematological recovery
DAC-CAG: Decitabine, aclacinomycin, cytarabine and granulocyte colony-stimulating factor regimen
DLI: Donor lymphocyte infusion
EFS: Event-free survival
ELN: European leukemia net
FLT3-ITD FLT3: Internal tandem duplication
G-CSF: Granulocyte colony-stimulating factor
GVHD: Graft-versus-host disease
GVL: Graft-versus-leukemia
HR: Hazard ratios
IFN-ɑ: Interferon-ɑ
IL-15: Interleukin-15
IQR: Interquartile range
MFC: Multi-parameter flow cytometry
MRD: Measurable residual disease
NR: Non-remission
OS: Overall survival
PCR: Polymerase chain reaction
VA: Venetoclax and azacytidine
VAH: Venetoclax, homoharringtonine and azacytidine
